# Forensic Application Evaluation of a Novel Canine STR System in Pembroke Welsh Corgi and Shiba Inu Groups

**DOI:** 10.1155/2021/5355109

**Published:** 2021-11-25

**Authors:** Yating Fang, Jinlong Yang, Yajun Deng, Bofeng Zhu

**Affiliations:** ^1^Guangzhou Key Laboratory of Forensic Multi-Omics for Precision Identification, School of Forensic Medicine, Southern Medical University, Guangzhou 510515, China; ^2^Multi-Omics Innovative Research Center of Forensic Identification, Department of Forensic Genetics, School of Forensic Medicine, Southern Medical University, Guangzhou 510515, China; ^3^Beijing Zhongzheng DNA Evidence Institute, Beijing 101318, China; ^4^Key Laboratory of Shaanxi Province for Craniofacial Precision Medicine Research, College of Stomatology, Xi'an Jiaotong University, Xi'an 710004, China

## Abstract

**Aim:**

To evaluate the forensic application values of 19 autosomal short tandem repeat (STR) loci in canines.

**Methods:**

The 19 STR loci in two canine groups (Pembroke Welsh Corgis, *n* = 200; Shiba Inus, *n* = 175) were analysed by the capillary electrophoresis platform. The allele frequencies and forensic parameters were calculated, and the genetic relationships between these two canine groups and a previously reported Labrador group were analysed.

**Results:**

These two canine groups conformed to the Hardy-Weinberg equilibrium at all STRs except for locus VGL3438 in the Pembroke Welsh Corgi group, and there was no linkage disequilibrium among pairwise loci at the 19 STRs. All STRs were polymorphic in the Pembroke Welsh Corgi and Shiba Inu groups, of which the locus C38 had the highest polymorphism. And it was found that the genetic relationship between the Pembroke Welsh Corgi and Labrador groups were closer in the three canine groups (Pembroke Welsh Corgi, Shiba Inu and Labrador).

**Conclusion:**

The 19 STR loci had high genetic polymorphisms and forensic application values in Pembroke Welsh Corgi and Shiba Inu groups.

## 1. Introduction

In recent years, it has been found that the study of non-human DNA genetic polymorphism has great significance for case investigation in the forensic practice, especially for canine which is closely related to human. Canine is the most frequently kept pet in the world today. Meanwhile, canine-related cases are increasing rapidly, such as cruelty to animal, attack on people or animal, involvement in crime scene, property damage, and the identification of lost pet. In 1999, Schneider et al. used mitochondrial DNA as case evidence that were extracted from canine hair [1]. In 2002, Padar et al. analysed short tandem repeats (STR) loci to detect a case involving a Hungarian canine which attacked a child to death [2]. In 2004, Wang et al. successfully extracted the canine DNA from the victim's mixed stain and genotyped by using the canine STR multiplex amplification technology in a vicious rape case involving domestic canine, and identified that the mixed stain contained the canine sperm. In 2016, Barrientos et al. reported a robbery and homicide case in which highly degraded DNA was extracted from canine stool samples [3]. In 2017, a fatal attack case was solved with canine genetic markers by Ciampolini et al. [4].

The analyses of DNA genetic polymorphisms are helpful for the identifications and pedigree controls of canine individuals. In 2011, the International Society for Forensic Genetics (ISFG) clarified that the non-human DNA analysis was similar to human DNA testing [5]. STRs are widely existing tandem repeat sequences with fragment length polymorphisms in the biological genome. It has been widely used in forensic identification and paternity testing. The STR analysis is not only low-cost and easy to operate in the capillary electrophoresis platform but also a widely popularized and highly utilized method in primary forensic DNA laboratories.

The molecular genetic markers in canine DNA also need to be evaluated in different groups like the genetic markers in human DNA. However, there are still relatively few studies on genetic polymorphisms and forensic application values of molecular genetic markers in different canine groups. Pembroke Welsh Corgis, the long-bodied and short-legged canines with erect ear and fox-like head, are the small canines originated from Wales of England. Shiba Inu is a kind of canine which has ancient origin from Japan. In this study, 200 purebred Pembroke Welsh Corgis and 175 purebred Shiba Inus were used as research objects to evaluate the genetic polymorphisms of 19 STR loci. It can not only assist in breeding pure Pembroke Welsh Corgis and Shiba Inus but also provide scientific evidences for the case investigations involving Pembroke Welsh Corgis or Shiba Inus in forensic caseworks.

## 2. Materials and Methods

### 2.1. Sampling and DNA Extraction

Saliva samples were taken from 200 purebred healthy Pembroke Welsh Corgis and 175 purebred healthy Shiba Inus based on the recommendations of the ISFG on non-human DNA analysis and the ethics committees of the Xi'an Jiaotong University Health Science and Southern Medical University. All canines were registered with the National General Kennel Club. Informed consents had been obtained from the owners of the canines before the study began. Genomic DNA was extracted by using the Chelex-100 method [6].

### 2.2. Amplification

The 19 autosomal STR loci (PEZ02, PEZ20, FH2010, FH2054, FH2001, vWF.X, FH2088, PEZ21, PEZ17, FH2328, FH2361, VGL2136, VGL3235, PEZ01, VGL3438, FH2004, C38, FH2611, and FH2137) of the commercial PBG Canine Genotype STR system (Beijing Protect Baby Gene Technology, China) were amplified in a 25 *μ*l system, which included 1 *μ*l DNA template, 5 *μ*l PCR buffer, 5 *μ*l Primer Mix, and 14 *μ*l sterile water. The amplification was performed on a GeneAmp® 9700 PCR thermocycler. The reaction conditions were as follows: initial denaturation at 95°C for 2 min, followed by 29 cycles at 94°C for 5 s and at 60°C for 1 min, and final incubation at 60°C for 10 min. And then, amplified products were held at 10°C.

### 2.3. Genotyping

After amplification, 1 *μ*l of the product or 1 *μ*l of ladder were combined with 9 *μ*l Hi-Di formamide and 0.3 *μ*l internal size standard. The 19 STR genotypes were conducted by capillary electrophoresis on Applied Biosystems™ 3500xL genetic analyser with default instrument settings. Subsequently, the raw data and the allelic ladder were analysed by GeneMapper ID-X software. All sizes were calculated by using internal size standards on the 70, 80, 100, 125, 150, 175, 200, 233, 266, 300, 333, 366, 400, 445, 490, and 500 bp.

### 2.4. Data Analyses

The Hardy-Weinberg equilibrium (HWE) and linkage disequilibrium (LD) were tested by the Arlequin software v3.5 [7] and the SHEsis online software [8], respectively. The allele frequencies and forensic parameters of STRs were calculated by the online software STRAF [9]. The inbreeding coefficient (*F*_IS_) was evaluated by genepop software v4 [10]. The principal component analysis (PCA) was performed by the R software v3.6.2 (https://cran.r-project.org/bin/windows/base/old/3.6.2/). The phylogenetic tree was conducted by MEGA X software [11]. The genetic structure was analysed by the STRUCTURE software v 2.3.4 [12], and the optimal *K* value was calculated by the online software Structure Harvester [13].

## 3. Results

### 3.1. Allele Frequency Distributions

A total of 165 alleles were observed at 19 STR loci in 200 purebred Pembroke Welsh Corgis with the allele frequency distributions from 0.0025 to 0.8325 (Table 1), and 180 alleles were in the Shiba Inu group with the allele frequency distributions from 0.0029 to 0.6371 (Table 2). In both the two groups, the allele distributions of locus C38 were the most extensive with a total of 18 alleles in the Shiba Inu group and 19 in the Corgi group (numbers of allelic repeat units were ranged from 10 to 37.1). In the 19 loci, locus FH2137 had the largest allele numbers with a total of 19 and 20 alleles in the Shiba Inu and the Pembroke Welsh Corgi groups, respectively. PEZ21 and vWF.X were the lowest number of alleles in the Pembroke Welsh Corgi group (only a total of 4), so were FH2010 and vWF.X in the Shiba Inu group.

### 3.2. HWE, LD, and *F*_IS_ Tests

In HWE tests, the correction level was adjusted to 0.0026 (0.05/19) after the Bonferroni correction. The estimated *P* values of all 19 loci were greater than 0.0026, suggesting that the two canine groups in this study were consistent with HWE except for locus VGL3438 in the Pembroke Welsh Corgi group.

**Figure 1:**
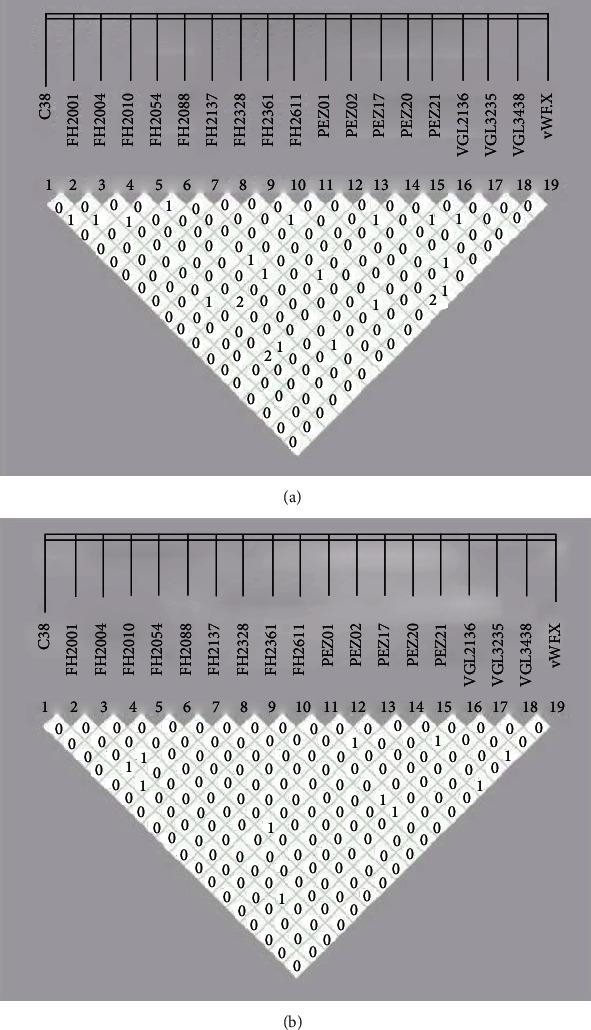
Linkage disequilibrium tests were conducted between pairwise STR loci in the studied canine groups. (a) Pembroke Welsh Corgi group (*n* = 200). (b) Shiba Inu group (*n* = 175). Linkage correlation coefficient (*r*^2^) values magnified 100 times were easy to be displayed in the small box.

Linkage correlation coefficient could be used to not only test the existence of LD but also evaluate the strength of LD. The correlation coefficient between the two loci was reflected by *r*^2^ values in this study. Figures 1(a) and 1(b) showed the *r*^2^ values of the linkage disequilibrium tests, in which 0 represented the value less than 0.01, 1 represented the value was from 0.01 to 0.02, and 2 represented the value was from 0.02 to 0.03. The results showed that all *r*^2^ values were less than 0.02, indicating that there was no linkage disequilibrium at the 19 STR loci in these two canine groups.

The *F*_IS_ was calculated to evaluate the level of inbreeding in these two canine groups. The *F*_IS_ value was 0.0619 in the Pembroke Welsh Corgi group, while the *F*_IS_ of the Shiba Inu group was 0.0865.

### 3.3. Forensic Parameters

**Figure 2:**
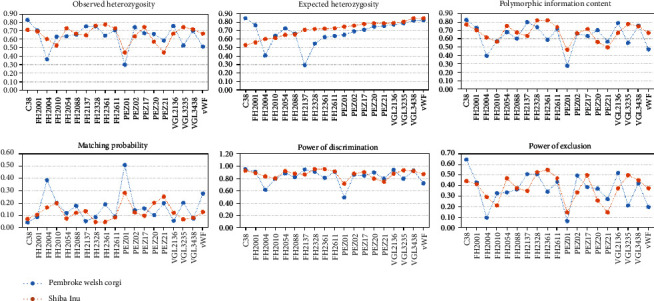
Forensic parameters for 19 short tandem repeat loci in the Pembroke Welsh Corgi group (*n* = 200) and the Shiba Inu group (*n* = 175).

As shown in Figure 2, the forensic application values of 19 STR loci in two canine groups were explored by calculating forensic parameters including matching probability (MP), polymorphic information content (PIC), observed heterozygosity (Ho), expected heterozygosity (He), power of discrimination (PD), and power of exclusion (PE).

In the corgi group, the Ho values of 19 STR loci ranged from 0.3000 to 0.8250, and the He values were from 0.2965 to 0.8426. The heterozygosity values (Ho and He) of loci PEZ01 and FH2004 were less than 0.5, and there were 7 loci (C38, VGL2136, FH2328, FH2137, PEZ02, FH2611, and FH2001) which were greater than 0.7. All PIC values were greater than 0.25, and 16 loci were greater than 0.5. The MP values ranged from 0.0450 to 0.5101, and the combined random match probability (CMP) was 3.04 × 10^−17^. The PD and PE values ranges from 0.4899 to 0.9551 and 0.0635 to 0.6462, respectively. The combined power of discrimination (CPD) and the combined power of exclusion (CPE) were 0.9999999999999999696 and 0.999889, respectively.

In the Shiba Inu group, the He values were a range from 0.5277 to 0.8428 with the average of 0.7166. The Ho values of all loci were greater than 0.5 (except for PEZ01 and PEZ21), so were the PIC values. The MP, PD, and PE values were in the range from 0.0475 to 0.2837, 0.7163 to 0.9525, and 0.1443 to 0.5471, respectively. The CMP, CPD, and CPE were 8.98 × 10^−19^, 0.999999999999999999102, and 0.999911, respectively.

### 3.4. Interpopulation Genetic Analyses

**Figure 3:**
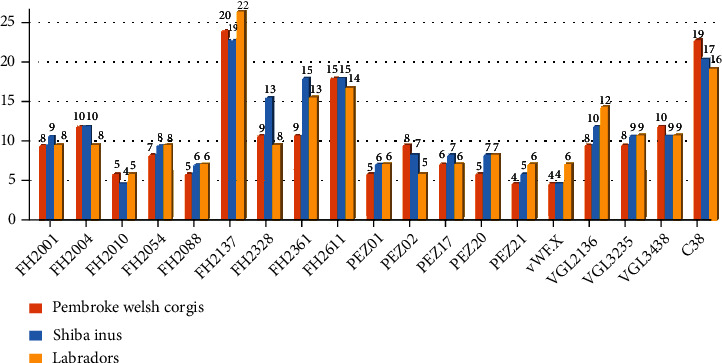
Comparison of the allele numbers between the studied two canine groups and the Labrador group based on 19 loci.

As shown in Figure 3, the differences of allele numbers at 19 STRs were compared among these two studied canine groups and a Labrador group (*n* = 214) [14]. The allele numbers of locus FH2137 were the largest in three canine groups, while the loci FH2010, FH2088, PEZ01, PEZ21, and vWF.X were relatively small.

**Figure 4:**
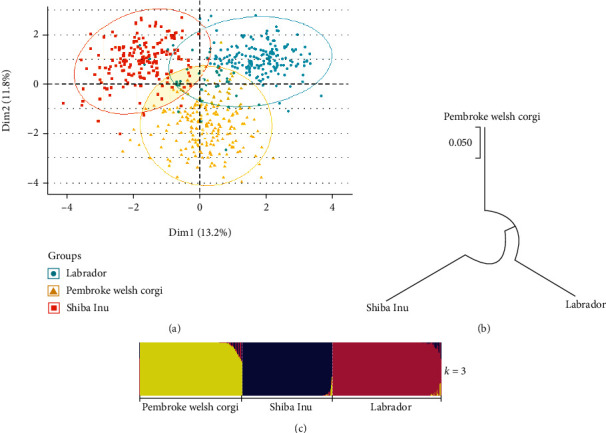
The genetic structures and genetic relationships of the Pembroke Welsh Corgi, Shiba Inu, and Labrador groups. (a) A clustering diagram of principal component analysis. (b) A phylogenetic tree. (c) A bar plot of structure analysis when the number of assumed populations (*K*) was 3.

The PCA and genetic structure analyses were performed to reveal the genetic structure of three canine groups. As shown in Figure 4(a), the clustering diagram of PCA divided the Pembroke Welsh Corgis, Shiba Inus, and Labradors into three clusters, and the small parts of three clusters overlapped with each other: Shiba Inus fell on the upper left of the axis, Labradors fell on the upper right, and Pembroke Welsh Corgis located on the lower. The numbers of assumed populations (*K*) were set to 2-7 in the structure analysis, and the optimal *K* value was determined to be 3 after calculations. As shown in Figure 4(c), the three canine groups had different ancestral components when *K* = 3. In Figure 4(b), a phylogenetic tree was conducted to understand the genetic relationships among these groups, and the results showed that the genetic relationship between the Pembroke Welsh Corgi group and the Labrador group was closer in all three groups.

## 4. Discussion

In this study, we investigated the genetic polymorphisms of 19 STR loci in 200 Pembroke Welsh Corgis and 175 Shiba Inus. Canine samples were collected, stored, and conducted in the manner that were similar with corresponding human forensic DNA analysis process, and the allele frequencies and forensic genetic parameters of the 19 loci in these two canine groups were evaluated, respectively, according to the recommendations of ISFG on non-human DNA analysis in forensic cases [5].

The present results showed that there was no linkage disequilibrium among the 19 STR loci, and only one locus (VGL3438) deviated from Hardy-Weinberg equilibrium in the corgi group, which may be the reason for the sampling. In the canine breeding process, inbreeding is often the most common way to pursue the purity of the breed. And canine pedigrees are usually not as well documented as those of humans, so it is difficult to establish the canine family tree. Therefore, the disturbance of the inbreeding still could not be completely avoided, although we tried to track breeding to avoid collecting the biological related samples in the sampling process, and exclude possible genetic related samples after comparing the genotyping results. How to circumvent this problem is still the difficulty of non-human DNA analysis. In addition, *F*_IS_ values were calculated to evaluate the level of inbreeding in this study, and the results showed the inbreeding levels of these two studied canine groups were low (0.0619 in the Pembroke Welsh Corgi group and 0.0865 in the Shiba Inu group), respectively.

In addition, the locus C38 with the highest heterozygosity, PIC, and PD values was high genetic diversities in the Pembroke Welsh corgi and Shiba Inu groups, which may have high potential in the applications of individual identifications of canines. The Pembroke Welsh Corgi, Shiba Inu, and Labrador groups had different genetic structures and a little genetic communications based on the results of PCA and structure analyses. The phylogenetic tree revealed that genetic relationship between the Pembroke Welsh Corgi group and Labrador group was closer in three canine groups. The Pembroke Welsh Corgi is an ancient breed of canine which originated from Wales, England. The modern Labrador is a king of breed that has been systematically bred and improved after being introduced into Britain in the 19th century, although they originally came from Newfoundland. The Shiba Inu is an ancient breed of Japanese origin. This may be the reason that the genetic relationship between the Pembroke Welsh Corgis and Labrador groups was closer than the genetic relationships between these two groups and the Shiba Inu group.

## 5. Conclusion

In this study, we found that the 19 STR loci have high genetic polymorphisms and forensic application values in the Pembroke Welsh Corgi and Shiba Inu groups. By genetic analysis among the two studied groups and the previously reported canine group, the Pembroke Welsh Corgi, Shiba Inu, and Labrador groups had different genetic backgrounds and structures, and the genetic relationship between the Pembroke Welsh Corgi and Labrador groups was relatively closer in three kinds of canine groups.

## Figures and Tables

**Table 1:** Allele frequencies for 19 short tandem repeat loci in the Pembroke Welsh Corgi group (*n* = 200).

Alleles	C38	Alleles	FH2004	Alleles	FH2137	Alleles	FH2361	Alleles	PEZ02	Alleles	VGL2136
14.2	0.1025	12	0.0250	17.3	0.0025	14	0.0250	10	0.0250	8	0.2875
15	0.0050	13	0.0575	18.3	0.0125	14.1	0.0025	11	0.0650	9	0.2125
15.2	0.0400	14	0.0400	19	0.0050	15	0.4750	13	0.0200	10	0.0975
16	0.1100	15	0.7650	19.3	0.0600	16	0.3425	14	0.1425	11	0.0100
16.2	0.0150	16	0.0300	20	0.0250	17	0.0900	15	0.3450	12	0.0800
17.2	0.3200	29	0.0200	20.2	0.0025	17.1	0.0025	16	0.3825	13	0.0925
18.2	0.0825	31	0.0300	20.3	0.3000	18	0.0250	17	0.0025	14	0.1825
19.2	0.1400	32	0.0150	21	0.0025	19	0.0350	18	0.0175	15	0.0375
20.2	0.0125	33	0.0100	21.3	0.1950	23	0.0025	*Alleles*	*PEZ17*	*Alleles*	*VGL3235*
21.2	0.0325	34	0.0075	22	0.0300	*Alleles*	*FH2611*	13	0.0025	12	0.0075
31.1	0.0025	*Alleles*	*FH2010*	22.3	0.1950	17	0.0025	14	0.4000	13	0.0200
33.1	0.0050	9	0.0075	23	0.0825	17.2	0.0025	15	0.1250	14	0.3800
34.1	0.0200	10	0.2875	23.2	0.0025	18	0.0475	16	0.3500	15	0.0750
35	0.0125	11	0.2300	23.3	0.0325	18.2	0.0300	17	0.1150	16	0.4775
35.1	0.0600	12	0.4725	24	0.0325	19	0.0325	18	0.0075	17	0.0100
36	0.0050	13	0.0025	24.3	0.0025	19.2	0.3625	*Alleles*	*PEZ20*	18	0.0100
36.1	0.0275	*Alleles*	*FH2054*	25	0.0025	20	0.0100	12	0.1600	19	0.0200
37	0.0050	16	0.0250	26	0.0075	20.2	0.0625	13	0.3750	*Alleles*	*VGL3438*
37.1	0.0025	17	0.2850	27	0.0025	21	0.0025	14	0.2250	12	0.2250
Alleles	*FH2001*	18	0.2575	28	0.0050	21.2	0.3250	15	0.1975	13	0.0050
8	0.0500	19	0.3475	*Alleles*	*FH2328*	22	0.0050	16	0.0425	14	0.0825
9	0.0725	20	0.0100	13	0.1225	22.2	0.0325	*Alleles*	*PEZ21*	15	0.0200
10	0.1125	21	0.0150	15	0.1775	23.2	0.0250	8	0.4925	16	0.1325
11	0.3850	23	0.0600	16	0.0025	24.2	0.0300	9	0.0150	17	0.3575
12	0.1075	*Alleles*	*FH2088*	17	0.0100	25.2	0.0300	10	0.2175	18	0.0125
13	0.2400	14	0.4250	18	0.3675	*Alleles*	*PEZ01*	11	0.2750	19	0.0525
14	0.0300	15	0.0175	19	0.0250	10	0.0050	*Alleles*	*vWF.X*	20	0.1025
14.2	0.0025	16	0.0975	20	0.0950	11	0.0175	9	0.6000	21	0.0100
		17	0.3725	21	0.1950	12	0.0850	10	0.2900		
		18	0.0875	22	0.0050	13	0.8325	11	0.0025		
						14	0.0600	12	0.1075		

**Table 2:** Allele frequencies for 19 short tandem repeat loci in the Shiba Inu group (*n* = 175).

Alleles	C38	Alleles	FH2004	Alleles	FH2137	Alleles	FH2361	Alleles	PEZ02	Alleles	VGL2136
10	0.0029	11	0.0029	17	0.0029	14	0.1229	12	0.0086	8	0.0229
11	0.0114	12	0.1029	17.3	0.0600	15	0.2429	13	0.4400	9	0.2343
14.1	0.0171	13	0.1771	18	0.0057	15.2	0.0343	14	0.0857	10	0.0029
15.2	0.1143	14	0.5429	18.3	0.0457	16	0.1714	15	0.2686	12	0.0029
16	0.0029	15	0.0571	19	0.0543	16.2	0.0029	16	0.1143	13	0.0514
16.2	0.0771	29	0.0057	19.1	0.0286	17	0.0829	17	0.0057	14	0.4229
17.2	0.0914	30	0.0943	19.3	0.0029	17.2	0.0029	18	0.0771	15	0.0086
18.2	0.4086	31	0.0086	20	0.0143	18	0.2057	*Alleles*	*PEZ17*	16	0.0343
19.2	0.0257	32	0.0057	20.1	0.0143	19	0.0686	14	0.1514	17	0.2171
20.2	0.0200	33	0.0029	20.3	0.0029	19.1	0.0086	15	0.2629	18	0.0029
26.1	0.0029	*Alleles*	*FH2010*	21	0.0143	19.2	0.0057	16	0.2343	*Alleles*	*VGL3235*
27.1	0.0029	9	0.1143	21.1	0.5743	20	0.0057	17	0.3057	12	0.2800
29.1	0.0229	10	0.2371	22	0.0114	20.1	0.0314	18	0.0343	13	0.2600
30.1	0.0800	11	0.0943	22.1	0.0686	20.2	0.0114	19	0.0029	14	0.1143
31.1	0.0914	12	0.5543	22.2	0.0029	21.1	0.0029	20	0.0086	15	0.0086
32.1	0.0257	*Alleles*	*FH2054*	23	0.0714	*Alleles*	*FH2611*	*Alleles*	*PEZ20*	16	0.0257
35.1	0.0029	17	0.0514	23.2	0.0057	18.2	0.0086	11	0.1514	17	0.1743
Alleles	*FH2001*	18	0.2000	24	0.0171	19.2	0.0657	12	0.0200	18	0.0543
6	0.0171	19	0.3514	24.2	0.0029	19.3	0.0229	13	0.5857	19	0.0771
8	0.1486	20	0.1714	*Alleles*	*FH2328*	20	0.0029	14	0.1714	20	0.0057
9	0.0114	21	0.0943	13	0.0114	20.2	0.1943	15	0.0571	*Alleles*	*VGL3438*
10	0.0171	22	0.1171	14	0.0029	21	0.0029	16	0.0114	11	0.1800
11	0.3143	23	0.0114	15	0.1543	21.2	0.3029	19	0.0029	12	0.3143
12	0.0314	24	0.0029	16	0.1657	22	0.0143	*Alleles*	*PEZ21*	13	0.1771
13	0.1086	*Alleles*	*FH2088*	17	0.0600	22.2	0.3000	9	0.0343	14	0.0200
13.2	0.3486	12	0.0029	17.1	0.0029	23	0.0229	10	0.0886	15	0.2257
14	0.0029	14	0.2314	18	0.0343	23.2	0.0314	11	0.6000	16	0.0657
Alleles	*PEZ01*	15	0.3771	18.1	0.2629	24	0.0057	12	0.2714	17	0.0086
9	0.0029	16	0.2543	19	0.1000	24.2	0.0029	13	0.0057	18	0.0057
11	0.6371	17	0.1286	19.1	0.0143	25	0.0200	*Alleles*	*vWF.X*	19	0.0029
12	0.0143	18	0.0057	20	0.0057	27	0.0029	9	0.1743		
13	0.2400			21	0.1514			10	0.3286		
14	0.1000			22	0.0343			11	0.3400		
15	0.0057							12	0.1571		

## Data Availability

The data underlying the findings of the study can be obtained by contacting the corresponding authors.
